# Elongation Patterns of the Collateral Ligaments After Total Knee Arthroplasty Are Dominated by the Knee Flexion Angle

**DOI:** 10.3389/fbioe.2019.00323

**Published:** 2019-11-12

**Authors:** Seyyed Hamed Hosseini Nasab, Colin R. Smith, Pascal Schütz, Barbara Postolka, Renate List, William R. Taylor

**Affiliations:** Laboratory for Movement Biomechanics, Institute for Biomechanics, ETH Zurich, Zurich, Switzerland

**Keywords:** collateral ligaments, elongation, dynamic fluoroscopy, gait, total knee arthroplasty

## Abstract

The primary aim of this study was to assess the effects of total knee arthroplasty (TKA) implant design on collateral ligament elongation patterns that occur during level walking, downhill walking, and stair descent. Using a moving fluoroscope, tibiofemoral kinematics were captured in three groups of patients with different TKA implant designs, including posterior stabilized, medial stabilized, and ultra-congruent. The 3D *in vivo* joint kinematics were then fed into multibody models of the replaced knees and elongation patterns of virtual bundles connecting origin and insertion points of the medial and lateral collateral ligaments (MCL and LCL) were determined throughout complete cycles of all activities. Regardless of the implant design and activity type, non-isometric behavior of the collateral ligaments was observed. The LCL shortened with increasing knee flexion, while the MCL elongation demonstrated regional variability, ranging from lengthening of the anterior bundle to slackening of the posterior bundle. The implant component design did not demonstrate statistically significant effects on the collateral elongation patterns and this was consistent between the studied activities. This study revealed that post-TKA collateral ligament elongation is primarily determined by the knee flexion angle. The different anterior translation and internal rotation that were induced by three distinctive implant designs had minimal impact on the length change patterns of the collateral ligaments.

## Introduction

The anterior and posterior cruciate ligaments are primary restraints in the natural knee (Boguszewski et al., 2011; Hosseini Nasab et al., 2016). Thus, after cruciate sacrificing Total Knee Arthroplasty (TKA), the passive restraint in the post-operative joint must be provided by the geometric congruency of the implant components and the surrounding soft tissues, predominantly the collateral ligaments. Thus, a thorough understanding of the relationships between postoperative ligament strains and implant design is crucial to improve future component geometries and soft tissue balancing techniques.

TKA joint stability has traditionally been achieved through concave depressions in the tibial insert that result in highly congruent interfaces with the medial and lateral femoral condyles (e.g., ultra-congruent designs). Posterior stabilizing designs attempt to provide additional stability through a post-cam mechanism, which constrains excessive posterior translation of the tibia relative to the femur. While both designs have been highly successful in achieving anteroposterior (AP) knee stability (Song et al., 2017), overly constrained knee motion has been observed clinically, especially regarding internal tibial rotation (Guan et al., 2017), and is thus a plausible cause of non-physiologic collateral ligament strain and possible pain.

Medial stabilized TKA component designs attempt to better replicate the kinematics of the natural knee by allowing substantial AP translation of the lateral condyle (Young et al., 2018). For example, the GMK Sphere (Medacta International, Switzerland) implant possesses a congruent spherical geometry on the medial side to provide anterior-posterior stability, and a relatively flat lateral tibial plateau to enable free movement of the lateral condyle. These intended implant motion patterns have indeed been confirmed through reconstruction of the 3D joint kinematics throughout both lunge movements and complete cycles of daily activities using mobile fluoroscopy (Scott et al., 2016; Schütz et al., 2019a). However, it is still not well-understood how such medially stabilized component designs affect the strains in the passive restraints of the knee.

The elongation patterns of the collateral ligaments during functional movements following TKA have important implications for post-operative knee stability, range of motion, and pain (Jeffcote et al., 2007; Babazadeh et al., 2009; Mihalko et al., 2009; Goudie and Deep, 2014). When ligaments are over-strained, cell death, plastic deformation, and micro-tears can occur (Provenzano et al., 2002). On the other hand, ligament unloading can lead to tissue adaptation and a diminished healing response to damage (Provenzano et al., 2003; Martinez et al., 2007). Thus, it is important for TKA component designs to restore normal elongation patterns of the collateral ligaments during functional activities.

The ability to estimate post-TKA ligament elongation patterns has critical implications for how to best balance the soft tissues. While adequate intraoperative balancing of the collateral ligaments during TKA is known to be essential for clinical success (Insall and Scott, 2001; Meloni et al., 2014), there is no consensus on what ligament fibers should be preserved and what fibers can be safely released. It is also not clear how the ligament balancing technique should be adapted based on the choice of implant design. Here, knowledge of elongation patterns of the individual ligament bundles in knees replaced with different implant designs could help surgeons to better understand the consequences of the release of specific ligament fibers and thereby enhance current soft tissue balancing approaches.

Although MRI and CT techniques have traditionally been used to provide access to quasi-static strain patterns, a number of methods exist to quantify ligament elongation during dynamic movements. Strain sensors have been applied to measure *in vivo* anterior cruciate ligament strains in patients undergoing partial meniscectomy (Fleming and Beynnon, 2004); however implantation and removal of the sensors is highly invasive and requires a wire to pass through the skin. Recently, an image-based approach was introduced that leverages fluoroscopy to quantify tibio-femoral kinematics, and then uses the relative movement of ligament attachment footprints to estimate elongation patterns. This approach has been utilized to measure anterior cruciate ligament elongation during stance in downhill running (Tashman et al., 2007), and collateral ligament elongations during the stance phase of walking (Liu et al., 2011) and a single legged lunge (Park et al., 2005; Van De Velde et al., 2007). However, the use of stationary fluoroscope setups has limited the range of dynamic movements that can be imaged.

Using a novel moving fluoroscope to overcome the limitations of a stationary imaging modality, this study aims to investigate the effects of TKA component design on collateral ligament elongation patterns that occur during level walking, downhill walking, and stair descent. Three component designs were evaluated: the GMK Primary posterior stabilized (PS), the GMK Sphere (SP), and the GMK Primary ultra-congruent (UC). In addition, the effect of activity type on ligament elongation patterns was investigated.

## Methods

**Figure 1:**
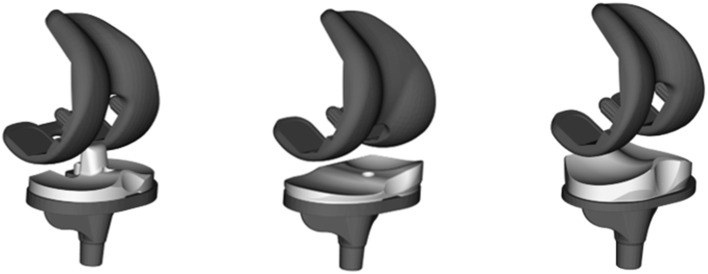
Three implant designs were investigated in this study: GMK Primary posterior stabilized **(left)**, GMK Sphere **(middle)**, and GMK Primary ultra-congruent design **(right)**.

Tibiofemoral kinematics were captured in three groups of patients with different TKA component designs (Figure 1) throughout complete cycles of activities of daily living. Each group consisted of 10 patients with unilateral TKA: the GMK Primary PS (5 m/5 f, aged 69.0 ± 6.5, 3.1 ± 1.6 years postoperative, BMI 27.6 ± 3.5), the GMK Sphere (2 m/8 f, aged 68.8 ± 9.9, 1.7 ± 0.7 years postoperative, BMI 25.4 ± 3.7) and the GMK Primary UC (3 m/7 f, aged 75.0 ± 5.1, 3.9 ± 1.5 years postoperative, BMI 25.9 ± 3.2). The study was approved by the local ethics committees (KEK-ZH-Nr. 2015-0140). Patients were selected based on the following inclusion criteria: unilateral TKA, more than 1 year after surgery, BMI ≤ 33, good clinical and functional outcomes (WOMAC between 0 and 28 and pain VAS ≤ 2). Kruskal–Wallis tests did not detect any statistically significant difference between the three groups regarding the age (*p* = 0.67), BMI (*p* = 0.81), time postoperatively (*p* = 0.27), or WOMAC score (*p* = 0.41) of the subjects. All the surgeries were performed by two senior knee surgeons using a medial parapatellar approach following the recommendations of the manufacturer. Surgeries were all performed with the aim to minimize soft-tissue damage. A mechanical hip-knee-ankle alignment of 180–183° was targeted. Where necessary, a very minimal release of the MCL was permitted in order to balance the knee, together with the removal of osteocytes that potentially blocked the smooth gliding of the soft tissues.

### Knee Joint Kinematics

**Figure 2:**
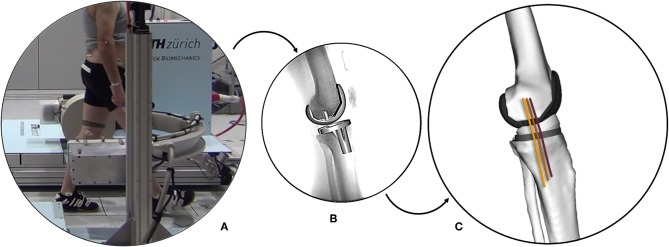
The ETH moving fluoroscope used to capture the knee joints during dynamic activities **(A)**. Implant component geometries registered to a fluoroscopic image **(B)**. Multi-body knee model including collateral ligament bundles **(C)**.

The moving fluoroscope (a modified Philips BV Pulsera video-fluoroscopy system mounted on a moving carriage; List et al., 2017; Hitz et al., 2018) at the Institute for Biomechanics, ETH Zürich was used to quantify tibio-femoral kinematics (Figure 2). Single plane fluoroscopic images were obtained at 25 Hz throughout five complete cycles each of level walking, downhill walking, and stair descent (Schütz et al., 2019a,b). In total, 450 gait cycles were measured (30 patients, 3 activities, each with 5 repetitions). For each imaging frame, the 3D pose of both the tibial and femoral implant components were determined using a 2D/3D registration algorithm introduced by Burckhardt et al. (2005) with an accuracy of up to 1° in rotation, 1 mm in-plane and 3 mm out-of-plane (Foresti, 2009).

### TKA Modeling

Tibial and femoral bone geometries, as well as the MCL and LCL attachment sites, were adapted from a previously developed multibody TKA knee model (Smith et al., 2016) within the OpenSim modeling environment (Delp et al., 2007). Subject-specific models were created by scaling each bone in the superior-inferior direction based on limb lengths measured using skin-mounted optical markers taken from reference standing trials (Vicon, OMG, Oxford, UK). Each model was additionally scaled in the anterior-posterior and medial-lateral directions based on the dimensions of the femoral implant.

**Figure 3:**
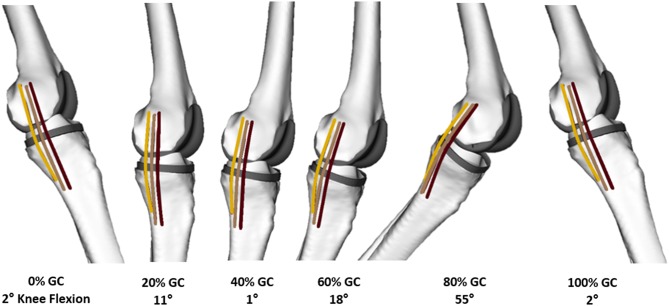
A medial view of the knee of a representative subject with the GMK Sphere implant at different instances throughout a level walking gait cycle (GC). Linear elements represent each of the anterior (dark brown), intermediate (light brown), and posterior (amber) bundles of the MCL.

The MCL and LCL were represented using a series of one-dimensional elements connecting their origin and insertion sites. The LCL was represented by a single fiber, while three fibers for the MCL corresponded to the anterior (aMCL), intermediate (iMCL), and posterior (pMCL) bundles of the ligament (Figure 3). Ellipsoidal wrapping objects were used to prevent penetration of the ligament bundles into the bone and implant geometries. For each of the 450 trials, the measured 3D implant kinematics were used as an input for the subject-specific OpenSim models to calculate the resulting ligament elongation patterns. The elongation of each ligament was normalized to its own reference length, which was defined as the average fiber length at heel strike of the five level walking trials (Liu et al., 2011), and presented against the normalized time in percentage of the gait cycle (%GC, starting with heel-strike) or the corresponding knee flexion angle.

### Statistical Analysis

For each implant design and activity, intra-subject variability of the ligament elongation was assessed by calculating the standard deviation (SD) between trials for a single subject at each time point of the activity cycle. These SDs were then averaged over the gait cycle and across subjects within an implant group.

A one-way repeated-measures ANOVA, based on statistical parametric mapping (SPM)—a statistical approach to examine differences in state-space or spatio-temporal data (Pataky et al., 2016)—was performed to test the influence of the implant design and activity on the ligament elongation patterns. Here, the test was considered significant if the maximum *F*-value (F_max_) from ANOVA exceeded the F_statistics_ (*F*-value corresponding to the significance level of *p* = 0.05).

To exclude the influence of between-subject variability of the ligament attachment points, the elongation patterns were further compared on the within-subject level. Therefore, for the three groups of implant designs, intra-subject differences in ligament elongations between the studied activities (absolute differences between the elongation patterns of different activities) were calculated at each specific flexion angle and results were averaged across subjects. These patterns were used to assess differences in ligament elongations between the designs and activities over the range of knee flexion angle.

### Sensitivity Analysis

To assess sensitivity of ligament elongation patterns to changes in secondary kinematic parameters, the pose of the tibia relative to the femur was perturbed around the baseline kinematics of a single gait cycle. Here, the anterior-posterior translation was perturbed within ±5 mm, while the abduction/adduction and internal/external rotations were varied within ±5°, using 1 mm and 1° intervals. It should be noted that only one of the input kinematic parameters was perturbed at a time and the other parameters were kept at their baseline values. For each parameter, the perturbed kinematics were fed into the multibody model of the corresponding subject and the output elongation patterns were plotted against time as percentage of the gait cycle. Moreover, to estimate the range of possible errors in ligament elongation outputs due to inaccuracy of the fluoroscopic kinematics, a worst-case scenario was simulated by perturbing the baseline kinematics captured from a subject with a PS implant design during level walking. Here, an out-of-plane error of 3 mm was introduced to the mediolateral translation. The perturbed kinematics were used to drive the multibody model of the corresponding subject and the output elongation patterns were plotted against time as percentage of the gait cycle.

## Results

### Implant Kinematics

A full description of the kinematics of the three implant designs has been reported previously (Schütz et al., 2019a), but are briefly summarized in Table S1 for completeness. All three implants showed similar ranges of knee flexion during the three studied activities with the GMK Primary UC exhibiting a slightly smaller range of motion compared with the other two implant designs. The GMK Sphere showed the smallest range of anteroposterior translation on the medial side for level walking, downhill walking, and stair descent, followed by the GMK UC and the GMK PS implants (SP was statistically significantly different from PS and UC, *p* < 0.006). The GMK Sphere showed the largest range of anteroposterior translation on the lateral side, as well as the largest range of tibial internal-external rotation (13.2 ± 2.2°), both observed during stair descent (SP was statistically significantly different from PS and UC, *p* < 0.006). All the three implant designs exhibited very limited range of abduction-adduction rotation during studied activities (average range smaller than 3°).

### Intra-subject Variability of the Ligament Elongations

**Figure 4:**
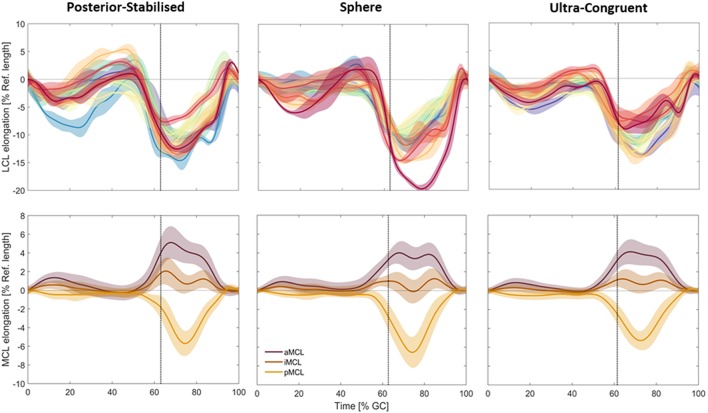
Subject-specific elongation patterns of the LCL (**top**, different color each subject, solid lines represent intra-subject means and shadings represent ± SDs) are shown compared to the average elongation patterns of the three MCL bundles (**bottom**, solid lines represent inter-subject means and shadings represent ± SDs) during level walking. The vertical dotted line represents the average toe-off time for subjects in the same group.

In general, the ligaments demonstrated relatively consistent elongation patterns across subjects and component designs throughout each activity (Figure 4). The largest intra-subject variations were observed in subjects with PS implants, while the smallest were observed in the UC implant subjects. Here, the average intra-subject standard deviation (SD) for the LCL elongation during level walking was 1.19, 1.08, and 1.05% for patients with PS, SP, and UC implants, respectively (*p* = 0.51). For comparison, the corresponding SDs for iMCL were 0.37, 0.33, and 0.29% (*p* = 0.10). Similar intra-subject variations were observed during downhill walking and stair-descent (Figures S1, S2), where the average within-subject SDs for LCL elongation were 1.18% (PS), 1.09% (SP), and 0.94% (UC) during downhill walking (*p* = 0.11) and 1.18% (PS), 1.07% (SP), and 0.98% (UC) during stair descent (*p* = 0.23).

The intra-subject variability of the ligament elongation was generally larger in the swing phase compared to the stance phase. For example, for the sphere implant, the average intra-subject standard deviation for LCL elongation during level gait was 1.80% for the stance and 2.99% for the swing phase.

### Effect of Implant Design

**Figure 5:**
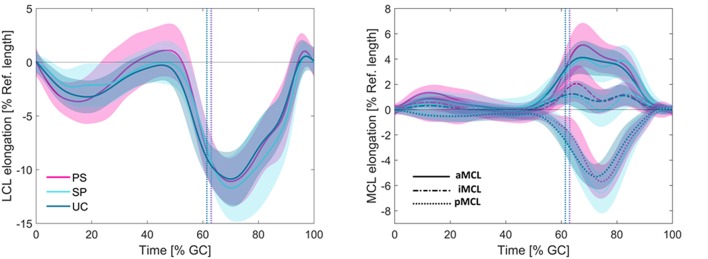
Average elongation patterns for the LCL **(left)** and MCL **(right)** during level walking. The vertical dotted lines represent the average toe-off times for the three groups. Solid lines represent inter-subject means and shadings represent ± SDs.

For all the three studied groups, slackening of the LCL was observed in the loading response period from 0 to 10% GC (Figure 5). From 10 to 50% GC, the ligament gradually recovered to its reference length. From push-off to mid-swing, the LCL experienced a rapid slackening and reached its shortest length (−11.09% in PS, −11.72% in SP, and −10.88% in UC; average across all subjects) at ~70% GC, which corresponded with peak knee flexion. As the knee was extended through terminal swing, the LCL continuously elongated to recover its reference length at the time of next heel strike.

Although the maximum shortening of the LCL was slightly greater in patients with sphere implants (Table S2), no statistically significant difference was observed between the elongation patterns of the LCL across the patient groups with differing component design (Table S3).

For downhill walking and stair decent, the LCL experienced a bi-phasic lengthening pattern consisting of steady shortening in the stance and a gradual stretching in the swing phase (Figures S1, S2). Compared to level walking, the LCL reached slightly shorter lengths during downhill walking and stair descent. The iMCL bundle was close to isometric during downhill walking and reached its shortest length at toe-off of stair-descent (−5.58, −6.27, and −5.94% of the reference length, for PS, SP, and UC groups; average across all subjects). Similar to the level walking, during downhill walking and stair descent, no statistically significant design-dependency was observed in the collateral ligament elongations (Table S3).

The length of the three MCL bundles remained nearly isometric during the first 50% of the level gait cycle. However, starting from late stance phase, the MCL bundles demonstrated considerably different elongation patterns (Figure 5). Regardless of the implant design, the anterior bundle experienced lengthening from 50 to 70% gait cycle with a maximum elongation of 5.11% for PS, 4.02% for SP, and 4.12% for UC implants. The posterior bundle showed the opposite elongation pattern, with shortening until 75% gait cycle, where it experienced its shortest length (−5.70% for PS, −6.56% for SP, and −5.29% for UC). The iMCL remained nearly isometric throughout the entire gait cycle, with the average elongation of each implant group never exceeding −0.29 and 2.06%.

Similar to level walking, non-uniform elongation of the MCL bundles was observed during downhill walking and stair-descent (Figures S1, S2). Regardless of the implant design, the aMCL experienced lengthening during the stance phase of downhill walking while the pMCL became shorter in this period. Except for 50–80% GC, the aMCL and pMCL had opposite elongation patterns during stair-descent. The iMCL bundle was close to isometric during downhill walking and was in its slackest condition at toe-off of the stair-descent (−5.58, −6.27, and −5.94% of the reference length, for PS, SP, and UC groups). Regardless of the activity, no significant design-dependency was observed in elongation patterns of the MCL bundles (Table S3).

The average range of length changes of the ligament bundles during the three studied activities were LCL: 11.1 ± 1.1%, aMCL: 4.4 ± 0.8%, iMCL: 3.7 ± 2.6%, and pMCL: 10.0 ± 4.3%.

### Effect of Activity Type

**Figure 6:**
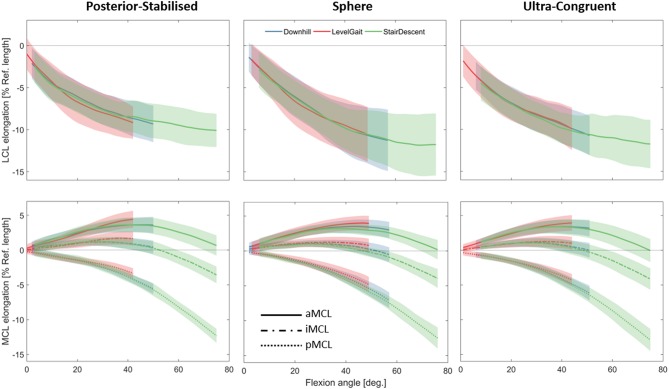
Average elongation of the LCL **(top)** and MCL **(bottom)** during five repetitions of the three studied activities plotted against the knee flexion angle. Average elongations were calculated only over the flexion ranges achieved by all the subjects during all the trials. Solid lines represent inter-subject means and shadings represent ± SDs.

In all three implant designs, shortening of the LCL and pMCL with increasing the knee flexion angle was observed during all the studied activities (Figure 6). The length of iMCL remained unchanged from full extension to 40–50° flexion and underwent a small shortening thereafter. During all the three activities, the aMCL lengthened from 0 to 40–50° flexion and shortened afterwards.

**Figure 7:**
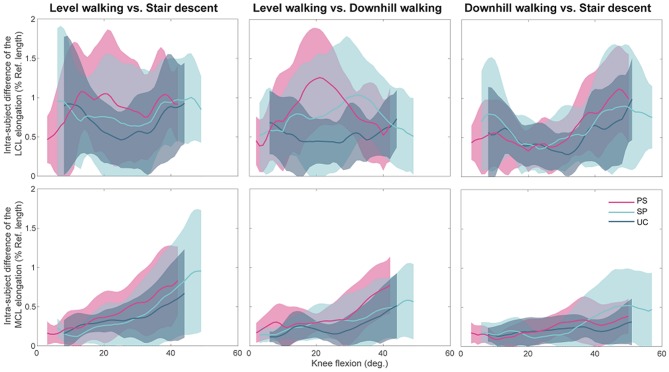
The intra-subject differences in ligament elongations between activities for the three groups of implant designs plotted against the knee flexion angle. Average patterns (solid lines) and standard deviations (shadings) were calculated only over the flexion ranges achieved by all the subjects within an implant design group during all the five included trials.

Regardless of the implant design, the statistical tests performed for assessing task-dependency of the ligament elongation were generally insignificant when comparing across patients with the same implant. This was consistent for the tests performed separately on the stance and swing phases of the activities (Tables S4, S5). However, the within-subject tests for task-dependency resulted in highly subject-specific outcomes. In some subjects the repeated measures ANOVA indicated significant differences between elongation patterns of the collateral ligaments during different activities. However, the mean-differences were generally small (compared to the corresponding standard deviations, Figure 7) and those differences were observed only over small ranges of flexion (Figure S3).

In general, all implant designs showed similar differences between the collateral ligament elongations when activities were compared (Figure 7). The only substantial difference between component designs was found in the comparison of LCL elongation between level and downhill walking (Figure 7, upper-middle), where the PS showed far greater differences than the UC. For each component design, the difference in MCL elongation between level-walking and stair descent showed a clear flexion dependency.

### Sensitivity of Ligament Elongations to Knee Kinematics

**Figure 8:**
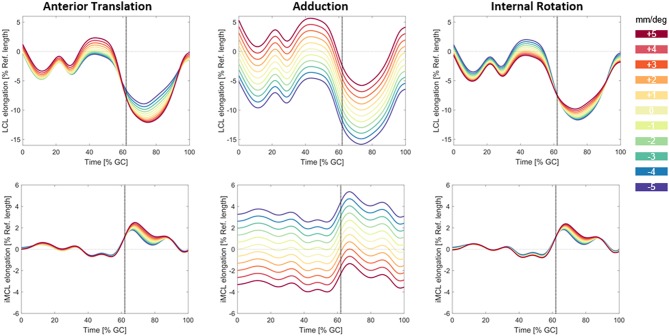
Variation in the LCL and iMCL elongation due to changes in anterior/posterior translation **(left)**, abduction/adduction rotation **(middle)**, and internal/external rotation **(right)** of the implant. The base line kinematics captured from a subject with PS design prosthesis during level walking was perturbed in the range of (±5 mm/±5°). The vertical dotted line shows the instant of Toe-off.

The highest impact of the changes in secondary kinematic parameters of the implant was due to variation in abduction/adduction parameter (Figure 8). During the stance phase of the gait cycle, a variation of ±5° in the adduction of the implant resulted in ±5.2% changes in the LCL elongation and ±3.5% of the iMCL elongation. A change of ±5 mm in the anterior translation of the implant had small influences on the ligament elongation patterns during level walking (about ±1.8% for LCL and ±0.5% for iMCL). A variation of ±5° in internal rotation of the implant had negligible influences on the elongation of collateral ligaments (about ±1.3% for LCL and ±0.4% for iMCL).

The simulated out of plane error (3 mm in the mediolateral translation) resulted in small changes in ligament elongation patterns (Figure S4). Here, the LCL was the most affected ligament with 0.53% change in the ligament elongation.

## Discussion

This study quantified elongation patterns of the collateral ligaments in patients with different TKA implant designs throughout complete cycles of level gait, downhill walking, and stair descent using kinematics measured by a novel moving fluoroscope to drive a multibody knee model with fiber based ligaments. Interestingly, the component design and activity type did not demonstrate statistically significant effects on the collateral elongation patterns. Instead, the knee flexion angle was the primary determinant of ligament elongation. The LCL shortened with increasing knee flexion, while the MCL demonstrated regional variability depending on the attachment location. The anterior MCL bundle lengthened from 0 to 40–50° and shortened thereafter, while the iMCL bundle remained isometric over the same rage, and then shortened with further flexion. The posterior MCL bundle demonstrated shortening with increased flexion across the entire range of motion. These results have important implications for surgeons performing intra-operative soft tissue balancing, and help provide a more comprehensive understanding of post-TKA ligament function during activities of daily living.

The presented ligament elongation patterns must be interpreted in the context of the limitations of the model. Since preoperative MRI and postoperative CT images were not available, the location of ligament attachments was approximated by scaling a generic model using the subject-specific implant dimensions and location of bony landmarks. However, even with high-resolution MR images, error on the order of centimeters remains in the assessment of the ligament attachment sites (Rachmat et al., 2014). We assessed the attachment location error using 10 fibers for the LCL and 20 fibers for the MCL with varying attachment locations and found the same general trends for the bundle elongation patterns as well as the design and activity dependencies. Moreover, the within-subject design that was used for assessing task-dependency of the ligament elongation patterns, minimizes the impact of uncertainty in ligament attachment points on the study results. Another limitation originates from one-dimensional path representations of the ligaments that may not reflect the actual elongations experienced by ligament fibers, as they do not account for material continuum, fiber twisting, or interfacial sliding. Similar to all image-based and many sensor-based studies, the zero-strain condition of the ligaments was not assessed in the current study. Thus, the elongation patterns reported in this study cannot be translated to the corresponding strain patterns, and therefore any between-study comparison of the data is limited to those with a consistent choice of reference length. Last but not least, the relatively small sample size and gender imbalance between the groups are clear limitations that should be addressed in future studies. In light of these limitations, the presented results should not be interpreted with a focus on the absolute magnitudes of the reported elongations, but rather on the differences between the ligament bundles, implant designs, and activities performed.

A key finding was that none of the ligament bundles showed purely isometric behavior during the studied activities for any of the implant designs. Only the iMCL during level walking demonstrated nearly isometric behavior. The LCL and pMCL demonstrated considerable shortening with increasing the knee flexion, while the aMCL was elongated at mid-flexion compared to full-extension and deep flexion. These findings provide *in vivo* evidence that the traditional intra-operative soft tissue balancing goal of achieving symmetrical flexion and extension gaps (Bottros et al., 2006; Daines and Dennis, 2014) does not reflect the dynamic function of the collateral ligaments. This is further supported by studies of dynamic limb alignment and internal-external rotation in intact knees that show increased laxity at 90° of flexion compared to extension (Roth et al., 2015). As such, our results support the recent trend in soft tissue balancing to aim for increased laxity in flexion compared to more extended poses (Roth and Howell, 2017).

A common cause for TKA patient dissatisfaction is mid-flexion instability (Hasegawa et al., 2018). This is often attributed to improper soft tissue balancing (Chang et al., 2014; Ramappa, 2015). Progressive release of the MCL is often used to correct varus alignment and achieve symmetric and rectangular gaps in 0 and 90° flexion (Griffin et al., 2000; Whiteside et al., 2000; Chen et al., 2011; Aunan et al., 2012). Our results demonstrate that the aMCL elongates with increasing flexion until 40–50°, with similar lengths at 0°, and 75° during stair descent, while the iMCL and pMCL shorten throughout the entire range of flexion. Thus, the aMCL likely provides the majority of the restraint at mid-flexion. Accordingly, any intraoperative release of the aMCL based on intraoperative laxity testing at 0 and 90° should be performed with caution. This supports previous research suggesting that over-release of the MCL is a plausible cause of mid-flexion instability of the replaced knees (Sharma, 2013; Ramappa, 2015) and that in addition to intraoperative laxity assessment at 0 and 90°, the tension balance should also be checked at mid-flexion (Bottros et al., 2006).

The flexion dependency of the collateral ligaments' elongations after TKA has previously been indicated by *in vitro* and *in vivo* experiments for simplified loading conditions, but it was uncertain whether this phenomenon extended to locomotor movements. In a cadaveric study (Ghosh et al., 2012), a differential variable reluctance transducer (DVRT) was sutured to the middle portion of the collateral ligaments after cruciate retaining TKA to quantify the MCL and LCL elongation patterns. Assuming average lengths of 100 mm (Liu et al., 2010) and 60 mm (Meister et al., 2000) for iMCL and LCL, they measured 2% slackening of iMCL and 12% slackening of the LCL at 60° knee flexion with an applied quadriceps force. Isometry of the iMCL after TKA was also reported by a similar cadaver study (Jeffcote et al., 2007). Interestingly, another *in vitro* investigation (Kowalczewski et al., 2015) found near isometry in the MCL and LCL during passive flexion, but during simulated squatting the LCL shortened and the MCL lengthened with increasing flexion. *In vivo* assessment of a forward lunge using stationary fluoroscopy revealed shortening of the LCL and pMCL, close to isometric behavior of iMCL and lengthening of the aMCL with increasing flexion (Park et al., 2015). Our results demonstrated similar elongation patterns to this *in vivo* study. Thus, our findings extend upon the previous literature to show that in multiple component designs and dynamic functional activities, the LCL shortens with increasing flexion, and the MCL elongation patterns vary between the anterior to posterior bundles until 40–50° flexion, after which all bundles shorten.

Despite small but significant differences in the *in vivo* measured kinematics (Schütz et al., 2019a), we found that posterior-stabilized, medial-stabilized, and ultra-congruent TKA designs all resulted in similar collateral elongation patterns (regardless of minor differences between their peaks) throughout complete cycles of level walking, downhill walking, and stair descent. The measured kinematics indicate that the GMK sphere implants indeed provide medial constraint, with the medial femoral condyle exhibiting a range of anterior-posterior translation of only 3.7 mm compared to 10.6 mm for PS, and 5.9 mm for UC implants during level walking. During stair descent, the GMK sphere also demonstrated the smallest range of medial anterior-posterior translation, as well as the largest range of internal/external tibial rotation (Sphere: 13.2°, PS: 9.0° UC: 8.4°). However, during all the studied activities, abduction/adduction was very consistent between the three implant designs (maximum difference of 0.7°). These kinematic differences due to implant design only resulted in subtle differences in ligament elongations. Here, the MCL and LCL elongations were most sensitive to perturbations in abduction/adduction (Figure 8), but the implant designs resulted in minimal kinematic variability in this degree of freedom. Anterior translation and internal/external rotation caused tibial movements that were perpendicular to the orientation of the collateral ligaments. These movements resulted predominantly in a gliding of the ligament, but only minimal length-changes of the collateral ligament fibers that are relatively long (100–120 mm for MCL), and therefore the resultant longitudinal strain was very small. Thus, as our sensitivity study demonstrates, a larger kinematic shift than what we observed along these degrees of freedom (DOF) is necessary to induce any detectable change in ligament elongation. As a result, the subtle kinematic differences measured between the component designs do not induce significant changes in MCL and LCL elongations.

While a potential goal to improve patient satisfaction for TKA is to restore the ligament elongation patterns in healthy knees, direct comparison of our results is limited by the available data on *in vivo* collateral ligament elongations during different functional activities. Using stationary fluoroscopy to study the stance phase of walking, Liu and co-workers found the aMCL elongated with increasing flexion, the iMCL remained relatively isometric, and the pMCL shortened with flexion (Liu et al., 2011). The magnitudes and trends of MCL elongation were generally similar to our measurements on TKA subjects during the stance phase of level walking. In a similar study examining single leg lunges, shortening of the MCL and the mid and posterior fibers of LCL was observed with increased knee flexion (Park et al., 2005). *In vitro* measurements of MCL and LCL elongation on intact cadaveric knees are widely available. Using DVRT sensors, Harfe and co-workers found the posterior fibers of MCL and LCL to be longest in extension and to shorten with knee flexion (Harfe et al., 1998). Gosh and co-workers measured the distance between the centers of ligament attachment sites and reported shortening of the superficial MCL and LCL during passive knee flexion from 0 to 110° flexion in the presence of a simulated quadriceps force (Ghosh et al., 2012). The elongation patterns presented in this study are therefore generally consistent in trend with those reported for the healthy knees at low flexion angles; however, none of the previous studies reported elongation data throughout complete cycles of level gait, downhill walking or stair decent.

## Conclusions

Understanding the postoperative elongation patterns of the collateral ligaments is crucial for improving intraoperative soft tissue balancing and implant design, in order to achieve better patient satisfaction following TKA. This study revealed that post-TKA ligament elongation is primarily determined by the knee flexion angle. The altered anterior translation and internal rotation that were induced by three different implant designs had minimal impact on the length change patterns of the collateral ligaments. Furthermore, the constrained geometries of the studied implant designs led to very similar flexion dependent MCL and LCL elongation patterns during level walking, downhill walking, and stair descent. However, generalization of our findings to other implant designs with distinctly different functionalities should only be undertaken with caution. Our results also have clinical implications for soft tissue balancing based on our observations of non-isometric behavior of the collateral ligaments with flexion. The conventional goal for ligament balancing techniques to achieve similar gaps at 0 and 90° flexion does not reflect the functional elongation patterns of the ligaments observed during activities of daily living. Improved techniques that account for differences in knee laxity and ligament elongation throughout the range of flexion should be considered.

## Data Availability Statement

The datasets generated for this study will not be made publicly available. Due to patient data privacy issues, these data can only be made available with an additional request to the ethics commission.

## Ethics Statement

The studies involving human participants were reviewed and approved by the local ethics committee of canton Zurich, Switzerland (KEK-ZH-Nr. 2015-0140). All participants provided written informed consent to participate in this study, and have allowed publication of their data in an anonymized manner.

## Author Contributions

SH was involved in planning of the study. He also processed the experimental data, performed the modeling and data analyses, and wrote the manuscript. CS helped in modeling, interpretation of the results, and editing the manuscript. PS and BP performed the measurements and helped in editing the manuscript. WT and RL were involved in planning and supervision of the study, interpretation of the data, and editing the manuscript. WT provided the resources used for experiments and simulations.

### Conflict of Interest

The authors declare that the research was conducted in the absence of any commercial or financial relationships that could be construed as a potential conflict of interest.
