# Supplementary material for: Elongation Patterns of the Collateral Ligaments After Total Knee Arthroplasty Are Dominated by the Knee Flexion Angle
Source: Front Bioeng Biotechnol. 2019 Nov 12;7:323. doi: 10.3389/fbioe.2019.00323 (PMC6861521; doi:10.3389/fbioe.2019.00323)
Supplement: Supplementary file 1 [file Data_Sheet_1.docx]

Supplementary Figures and Tables:


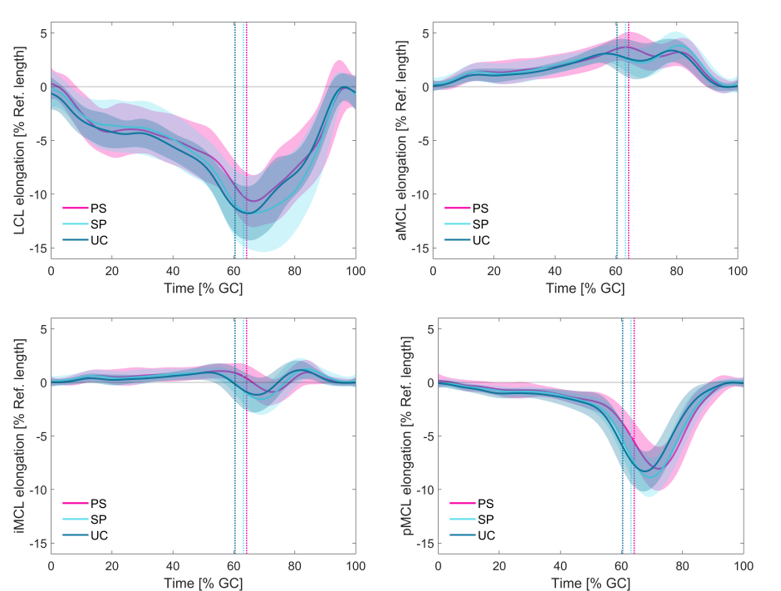


**Figure S1.** Average elongation patterns for the LCL and MCL bundles during downhill walking. The vertical dotted lines represent the average toe-off times for the three groups. Solid lines represent inter-subject means and shadings represent ±SDs.


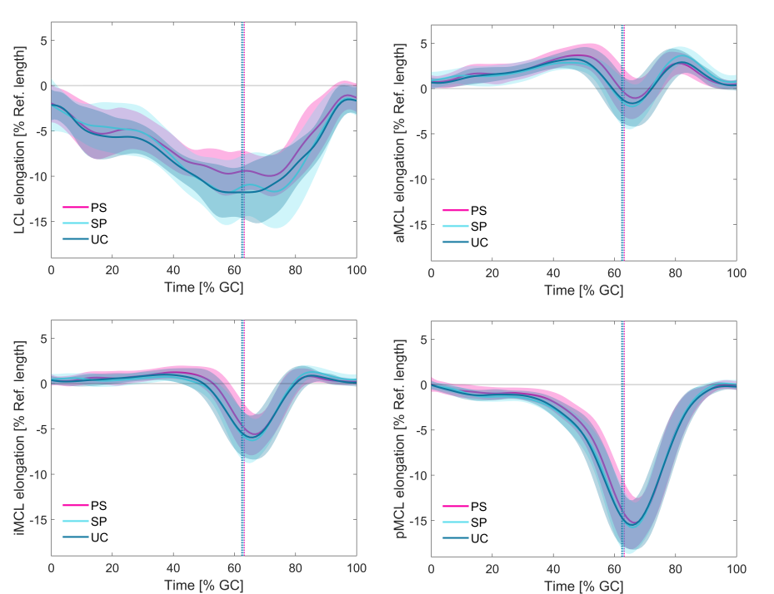


**Figure S2.** Average elongation patterns for the LCL and MCL bundles during stair descent. The vertical dotted lines represent the average toe-off times for the three groups. Solid lines represent inter-subject means and shadings represent ±SDs.


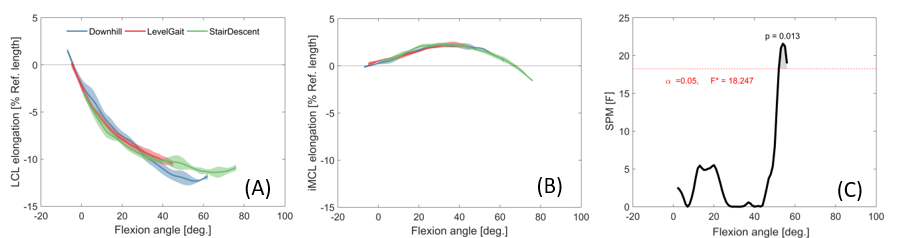


**Figure S3.** Elongation of the LCL and iMCL (A and B) during stance phase of the studied activities in a representative subject with a sphere implant (Solid lines represent intra-subject means and shadings represent ±SDs). The SPM plot (C) shows the SPM {F} for the test performed to assess task-dependency of the LCL elongation (level walking vs. downhill walking). The red dashed line represents the critical threshold corresponding to a significance level of α = 0.05 and the shaded grey area shows the region where the statistical test for task-dependency was significant (p=0.013).


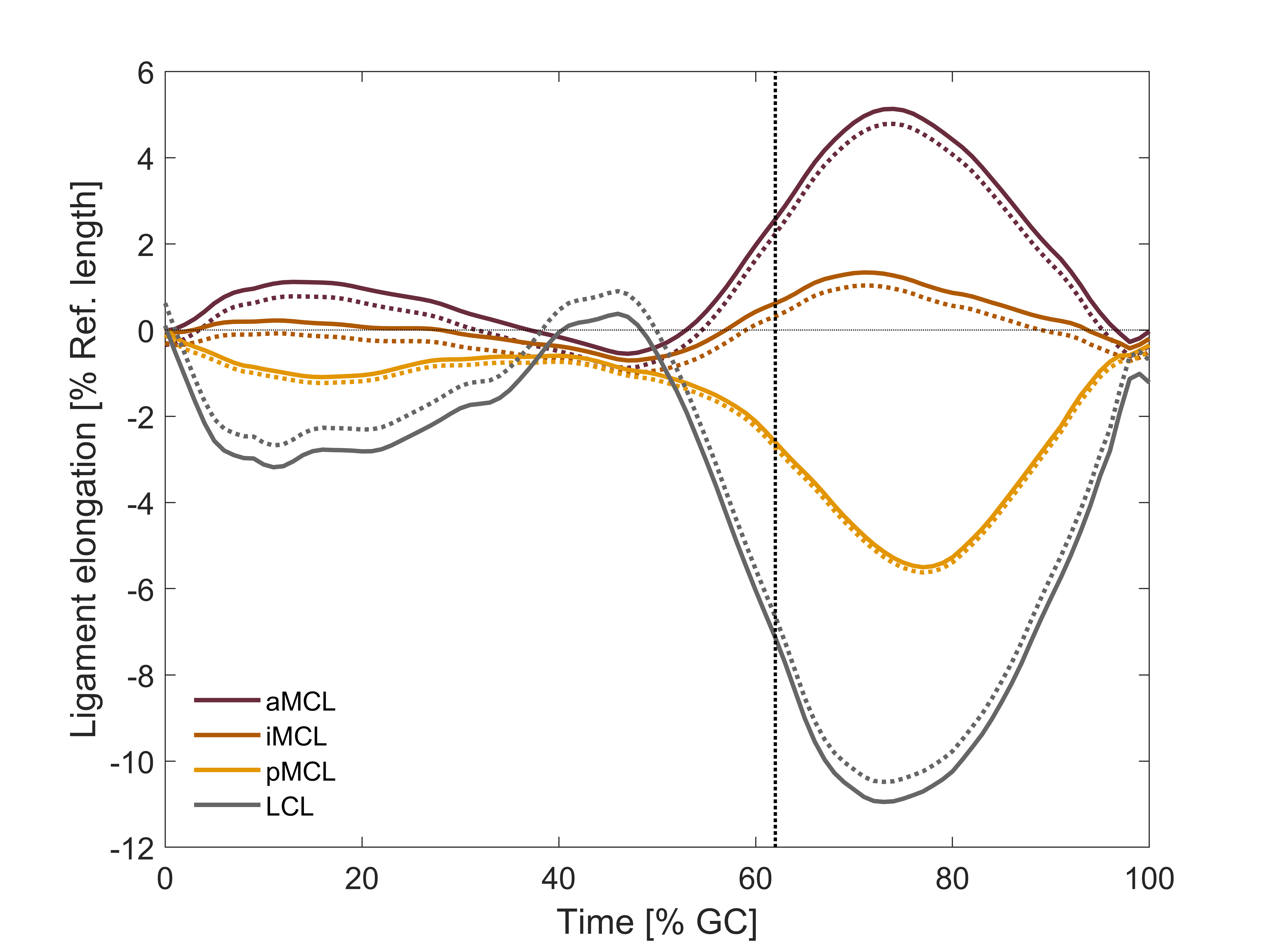


**Figure S4.** Variation in the LCL and MCL elongation due to a simulated out-of-plane error. The baseline kinematics captured from a subject with PS design prosthesis during level walking was perturbed by 3 mm in the mediolateral direction. The vertical dotted line shows the instant of Toe-off.

**Table S1.** Summary of the implant kinematics (range of motions).

|  | Flexion-Extension (°) | | | Antero-Posterior translation of articular contact point (mm) | | | | | |
| --- | --- | --- | --- | --- | --- | --- | --- | --- | --- |
|  | PS | SP | UC | PS | | SP | | UC | |
|  |  |  |  | Medial | Lateral | Medial | Lateral | Medial | Lateral |
| Level Walking | 63.5±4.7 | 62.7±4.9 | 57.2±4.8 | 10.3 ± 2.2 | 8.4 ± 1.6 | 3.6 ± 0.9 | 10.6 ± 4.4 | 5.7 ± 1.0 | 5.5 ± 1.4 |
| Downhill Walking | 69.9±5.3 | 70.0±4.5 | 66.1±3.4 | 10.1 ± 2.6 | 9.1 ± 2.3 | 3.1 ± 0.8 | 9.9 ± 3.0 | 8.0 ± 1.7 | 5.7 ± 1.1 |
| Stair Descent | 90.2±5.5 | 89.5±5.5 | 87.5±4.4 | 11.6 ± 1.6 | 10.7 ± 2.6 | 3.9 ± 1.3 | 12.1 ± 2.2 | 8.7 ± 1.9 | 6.9 ± 1.6 |

**Table S2.** Summary of the ligament elongation data (averaged per group).

| **Design** | **Ligament** | **Downhill Walking** | | | **Level Walking** | | | **Stair Descent** | | |
| --- | --- | --- | --- | --- | --- | --- | --- | --- | --- | --- |
|  |  | Max. elong. | Min. elong. | Range elong. | Max. elong. | Min. elong. | Range elong. | Max. elong. | Min. elong. | Range elong. |
| **PS** | LCL | 0.29 | -10.65 | 10.94 | 1.13 | -11.09 | 12.22 | -1.06 | -9.96 | 8.91 |
|  | aMCL | 3.67 | 0.01 | 3.66 | 5.11 | -0.28 | 5.4 | 3.67 | -1.05 | 4.72 |
|  | iMCL | 1.06 | -0.89 | 1.95 | 2.06 | -0.29 | 2.34 | 1.24 | -5.58 | 6.83 |
|  | pMCL | 0.16 | -8.07 | 8.23 | 0.27 | -5.70 | 5.97 | 0.02 | 15.24 | 15.27 |
| **SP** | LCL | 0.03 | -11.75 | 11.78 | 0.63 | -11.72 | 12.36 | -1.41 | -11.80 | 10.40 |
|  | aMCL | 3.83 | 0.06 | 3.77 | 4.02 | -0.02 | 4.04 | 3.65 | -1.97 | 5.62 |
|  | iMCL | 1.25 | -1.57 | 2.82 | 1.27 | 0.11 | 1.38 | 1.28 | -6.27 | 7.55 |
|  | pMCL | 0.07 | -8.85 | 8.92 | 0.07 | -6.56 | 6.63 | 0.13 | -15.77 | 15.90 |
| **UC** | LCL | -0.03 | -11.78 | 11.75 | 0.56 | -10.88 | 11.44 | -1.53 | -11.79 | 10.26 |
|  | aMCL | 3.37 | -0.04 | 3.41 | 4.12 | 0.09 | 4.21 | 3.24 | -1.63 | 4.87 |
|  | iMCL | 1.51 | -1.16 | 2.31 | 1.25 | -0.2 | 1.45 | 0.99 | -5.94 | 6.93 |
|  | pMCL | 0.03 | -8.30 | 8.27 | 0.12 | -5.29 | 5.41 | -0.04 | -15.49 | 15.45 |

**Table S3.** F_max_ and F-statistics (in bracket) for the SPM tests performed to assess design-dependency of the ligament elongation patterns.

|  | **Level Walking** | | | **Downhill Walking** | | | **Stair Descent** | | |
| --- | --- | --- | --- | --- | --- | --- | --- | --- | --- |
|  | PS vs. SP | SP vs. UC | PS vs. UC | PS vs. SP | SP vs. UC | PS vs. UC | PS vs. SP | SP vs. UC | PS vs. UC |
| **LCL** | 11.30 (20.15) | 12.47 (19.89) | 6.97 (20.26) | 4.82 (19.64) | 13.24 (19.33) | 10.89 (19.60) | 2.00 (19.45) | 1.12 (19.58) | 7.29 (19.65) |
| **aMCL** | 7.49 (21.04) | 11.44 (20.72) | 8.46 (21.18) | 2.76 (21.50) | 5.75 (21.40) | 9.35 (21.35) | 4.84 (21.47) | 5.29 (21.42) | 3.22 (21.32) |
| **iMCL** | 4.90 (22.00) | 4.28 (21.55) | 6.00 (22.05) | 5.93 (22.27) | 3.87 (22.19) | 20.04 (22.34) | 6.65 (21.96) | 9.34 (21.91) | 3.46 (21.97) |
| **pMCL** | 8.78 (21.97) | 10.87 (21.48) | 17.18 (21.56) | 10.80 (21.17) | 4.48 (21.18) | 14.12 (21.08) | 5.69 (21.28) | 20.01 (21.12) | 3.24 (21.40) |

**Table S4.** Fmax and F-statistics (in bracket) for the SPM tests performed to assess task-dependency of the ligament elongation patterns during stance phase of the studied activities.

|  | **PS** | | | **SP** | | | **UC** | | |
| --- | --- | --- | --- | --- | --- | --- | --- | --- | --- |
|  | LW vs. DW | LW vs. SD | DW vs. SD | LW vs. DW | LW vs. SD | DW vs. SD | LW vs. DW | LW vs. SD | DW vs. SD |
| **LCL** | 1.27 (8.38) | 5.76 (30.85) | 3.60 (11.06) | 0.20 (8.15) | n/a | 2.22 (12.38) | 0.49 (7.88) | 0.63 (7.16) | 2.74 (11.56) |
| **aMCL** | 9.09 (8.21)* | 3.47 (30.85) | 4.43 (10.95) | 5.21 (7.97) | n/a | 6.79 (12.64) | 1.74 (7.82) | 0.71 (7.12) | 1.69 (11.74) |
| **iMCL** | 4.63 (8.18) | 0.17 (30.85) | 4.08 (11.16) | 4.16 (7.90) | n/a | 6.75 (12.61) | 0.28 (7.87) | 0.18 (7.08) | 1.03 (11.82) |
| **pMCL** | 3.72 (8.16) | 0.08 (30.85) | 4.87 (11.18) | 3.29 (7.85) | n/a | 6.33 (12.68) | 0.22 (7.92) | 0.23 (7.10) | 0.91 (11.88) |

* indicates a significant difference (α ≤ 0.05). LW, DW and SD represent level walking, downhill walking and stair decent.

**Table S5.** F_max_ and F-statistics (in bracket) for the SPM tests performed to assess task-dependency of the ligament elongation patterns during swing phase of the studied activities.

|  | **PS** | | | **SP** | | | **UC** | | |
| --- | --- | --- | --- | --- | --- | --- | --- | --- | --- |
|  | LW vs. DW | LW vs. SD | DW vs. SD | LW vs. DW | LW vs. SD | DW vs. SD | LW vs. DW | LW vs. SD | DW vs. SD |
| **LCL** | 4.87 (10.55) | 14.35 (11.16)* | 2.95 (10.98) | 0.43 (12.58) | 3.18 (11.96) | 5.67 (12.09) | 8.91 (11.45) | 1.03 (11.25) | 5.14 (11.55) |
| **aMCL** | 29.76 (10.61)* | 31.76 (12)* | 9.07 (10.36) | 9.34 (11.81) | 56.92 (11.43)* | 31.23 (11.98)* | 6.07 (11.34) | 6.58 (11.07) | 2.85 (11.59) |
| **iMCL** | 19.80 (10.27)* | 17.97 (11.69)* | 3.05 (10.30) | 8.08 (11.27) | 52.55 (11.32)* | 29.81 (11.93)* | 2.06 (11.30) | 3.48 (11.00) | 2.89 (11.75) |
| **pMCL** | 17.58 (10.21)* | 15.30 (11.61)* | 2.23 (10.36) | 8.44 (11.71) | 43.76 (11.32)* | 26.53 (11.91)* | 2.01 (11.34) | 3.09 (10.99) | 2.96 (11.84) |

* indicates a significant difference (α ≤ 0.05). LW, DW and SD represent level walking, downhill walking and stair decent.
